# Levels of whole salivary advanced glycation end products and interleukin-17 and peri-implant clinical and radiographic status in patients with osteoporosis at 6-years’ follow-up

**DOI:** 10.1186/s12903-022-02591-7

**Published:** 2022-11-24

**Authors:** Fawaz Al-Qahtani, Nasser Alqhtani, Darshan Devang Divakar, Fahad Alkhtani, Firas K. Alqarawi

**Affiliations:** 1 Department of Prosthodontics, School of Dentistry, Prince-Sattam Bin Abdulaziz University, Al-Kharj, 11942 Saudi Arabia; 2 Department of Oral and Maxillofacial Surgery and Diagnostic Sciences, College of Dentistry, Prince Sattam Bin Abdulaziz University, Al-Kharj, 11942 Saudi Arabia; 3 Department of Dental Medicine, Sharavathi Dental College and Hospital, Shivamogga, Karnataka 577204 India; 4 Department of Prosthodontics, College of Dentistry, Prince Sattam Bin Abdulaziz University, 11942, Al-Kharj, Saudi Arabia; 5 Department of Substitutive Dental Sciences, College of Dentistry, Imam Abdulrahman Bin Faisal University, P.O. Box 1982, Dammam, 31441 Saudi Arabia

**Keywords:** Advanced glycation endproducts, Alveolar bone loss, Osteoporosis, Probing depth

## Abstract

**Background:**

There are no studies that have assessed advanced glycation end products (AGEs) and interleukin 17A (IL-17A) levels in whole saliva (WS) of patients with dental implants. The aim was to compare levels of AGEs and IL-17A in WS and peri-implant clinical and radiographic status of patients with and without osteoporosis at 6-years’ follow-up.

**Methods:**

Osteoporotic (Group-1) and systemically healthy controls (Group-2) having undergone dental implant therapy at least 5 years ago were included. A questionnaire was used to collect information about age, gender, duration and treatment of osteoporosis, number and duration of implants in function, and frequencies of toothbrushing, flossing and hygiene visits. Modified bleeding and plaque indies (mBI and mPI), peri-implant probing depth (PD) and crestal bone loss (CBL) were recorded. WS was collected and levels of AGEs and IL-17A were determined using enzyme linked immunosorbent assay. Sample-size estimation was done and statistical analyses were doing using the independent t- and Wilcoxon rank-sum tests. Statistical significance was marked for P-values that were below 0.01.

**Results:**

In patients with (n = 24) and without (n = 27) osteoporosis, implants were in function for 6.3 ± 0.27 and 6.6 ± 0.5 years, respectively. There was no significant difference in peri-implant mBI, PD, mPI and CBL in both groups. Levels of AGE in patients with (119.6 ± 26.5 μg/ml) and without (91.5 ± 14.6 μg/ml) osteoporosis were comparable. Levels of whole salivary IL-17A in patients without and with osteoporosis were 4.6 ± 0.3 and 5.1 ± 0.8 pg/ml, respectively. Flossing of full mouth interproximal spaces once and twice daily toothbrushing daily was reported by 100% patients with osteoporosis. Among patients with osteoporosis 75% and 25% individuals were receiving professional dental prophylaxis every 4 and 6 months, respectively.

**Conclusion:**

Within the limitations of the present study osteoporotic patients are not at an increased risk of peri-implant diseases and can demonstrate salivary AGE and IL-17A levels comparable to non-osteoporotic individuals as long as oral hygiene is stringently maintained.

**Supplementary Information:**

The online version contains supplementary material available at 10.1186/s12903-022-02591-7.

## Introduction

Osteoporosis is an osseous disorder characterized by decreased bone density or low bone mass and structural deterioration of osseous tissues. This makes bones vulnerable to pathological fracture [1]. Osteoporosis has been categorized into two main types: primary (most common form and often manifested in post-menopausal phase) and secondary (characterized as having a defined etiologic mechanism) [1]. From an immunoinflammatory perspective, glycotoxins or advanced glycation endproducts (AGE) are recognized as mediators of the development and pathogenesis of osteoporosis [2]. These endproducts are formed by non-enzymatic oxidation of proteins and lipid [3–5]; however, their production is significantly increased under a state of oxidative stress and/or inflammation. Accumulation of AGE within osseous tissues induces the formation of covalent cross-links with collagen and other proteins that jeopardizes osseous mechanical properties and disturbs bone remodelling [2]. The AGE also activate the formation of destructive inflammatory cytokines such as tumor necrosis factor alpha (TNF-α), interleukin (IL)-6, IL-10, and IL-17A that facilitate and accelerate osteoclastic activity [6, 7]. Bisphosphonates are often used for the management of osteoporosis [8].

Dental implants (DI) can present survival rates ranging up to 100% [9, 10]; however, complications and failures in relation to DI therapy can happen in certain situations. A dilemma in clinical implant dentistry is the occurrence of peri-implantitis which if left untreated in a timely manner can result in extensive crestal bone loss (CBL) and even implant failure [11, 12]. Studies [13–15] have shown that osteoporosis compromises long-term survival of dental implants. According to Giro et al. [16] rate of implant failure is nearly 11% in patients with osteoporosis. In addition, the risk of development of ONJ is a serious consideration before and after DI placement and loading in patients undergoing BT, such as osteoporotic patients [17–19]. Moreover, bone-to-implant contact is compromised by approximately 50% in patients with osteoporosis. Nevertheless, a consensus as to whether or not osteoporosis is a risk-factor of peri-implant diseases is yest to be established [20]. An increased expression of AGE in whole saliva (WS), gingival crevicular fluid (GCF) and peri-implant sulcular fluid (PISF) has been linked with the etiopathogenesis of peri-implant diseases [21, 22]. Raised levels of AGE have been identified in the PISF of patients with peri-implantitis [21–23]. Similarly, IL-17A levels have also been identified in the PISF of patients with peri-implant diseases [24, 25]. There are however no studies that have assessed AGE and IL-17A levels in WS of patients with DI. A review of indexed literature also showed no studies that assessed whole salivary IL-17A and AGE levels among patients with and without osteoporosis.

The aim was to compare levels of AGEs and IL-17A in WS and peri-implant clinical and radiographic status of patients with and without osteoporosis at 6-years’ follow-up. The present study was based on the *null* hypothesis that there is no difference in whole salivary AGE and IL-17A levels and peri-implant clinical and radiographic status among patients with and without osteoporosis.

## Methods

### Ethical guidelines

The present investigation study was approved by the human subjects ethics board of the Sharavathi Dental College and Hospital, Shivamogga, Karnataka 577,204, India (21/2021-CR); and was carried out in accordance with the Helsinki Declaration of 1975, as revised in 2013. Participation was completely voluntary; and patients reserved the right to withdraw their participation at any stage of the study. There were no penalties associated with withdrawal. All patients were mandated to sign a consent form prior to inclusion in the present study.

### Inclusion and exclusion criteria

The inclusion criteria were as follows: (a) patients that had DI-retained prosthesis for at least 5-years; (b) patients with medically diagnosed osteoporosis; (c) controls (individuals without a medical diagnosis of osteoporosis). Osteoporotic patients were defined as those with an overall bone mineral density (BMD) score of <  − 2.5 [26]. An overall BMD score of between -2.5 and -1.0 was evidence of osteopenia [26]. An overall BMD score over -1 was categorized as “normal” [26]. Self-reported tobacco product consumers, patients with other systemic diseases such as diabetes mellitus (DM), respiratory diseases and cardiovascular disorders (CD) and patients that had used antibiotics, and steroids within 90 days were excluded.

### Invitation letter, information sheet and consent form

Patients with and without a history of osteoporosis that had undergone DI treatment for rehabilitation of missing teeth were recruited from the dental department of a local healthcare institution in Karnataka, India. Diagnosis of osteoporosis was verified from the patients’ medical records. Written invitation letters were posted to individuals with and without a diagnosis of osteoporosis along with an information sheet that described the purpose of the present study in simple words. A consent form was also posted and consenting individuals were requested to bring a signed version of the consent form to the Dental department of Sharavathi Dental College and Hospital, Shivamogga, Karnataka 577,204, India.

### Questionnaire

Demographic information (sex, age in years, ethnic origin and gender) and information regarding daily toothbrushing and interproximal flossing was recorded. Participants were also asked how often they went to a dentist or hygienist for dental prophylaxis.

### Assessment of medical and dental records

Duration since diagnosis of osteoporosis and its treatment was retrieved from patients’ medical records. Information regarding DI characteristics (number of DI and their location in jaws, design, diameter and length, and mode of prosthesis retention) and duration for which, DI were functional was extracted from patient’s dental records.

### Clinical and radiologic investigations

Modified bleeding and plaque indices (mBI [27] and mPI [27, 28]), and probing depth (PD) [29, 30] were measured using standard techniques. The CBL was measured on digital bitewing x-rays using a software program (Image Tool 3.0 Program, Department of Dental Diagnostic Science, University of Texas Health Science Center, San Antonio, TX, USA) as the linear distance from two-millimeters below the implant abutment interface to the crestal bone [31]. All clinical and radiographic investigations were done by a calibrated and blinded examiner (Kappa score 0.86).

### Saliva collection and determination of levels of AGE and IL-17A

The WS samples were collected during early morning hours (between 7:30 am and 8:30 am) with the participants being in a fasting state. Saliva collection was done 24 h after clinical and radiographic assessments. The unstimulated WS was collected and salivary flow rate was determined using enzyme linked immunosorbent assay as described in other studies [32, 33]. Immediately upon WS collection and determination of flow rate, all samples were centrifuged at 1000 × g in a cold room at 4 °C for 15 min and the supernatant was collected. The supernatant was stored at − 80 °C until further analysis. All WS supernatants were assessed for AGE and IL-17A levels within 24 h of collection. Levels of AGE were quantified using a commercial AGE kit (Oxiselect, Cell Biolabs, Inc., San Diego, CA, USA) in accordance with the protocol described in the study by Katz et al. [32] Briefly, each supernatant sample was diluted in phosphate buffered saline (PBS) in a 1:1 ratio. Spectrofluorometric analysis using a plate reader (BioTek Synergy HT, Winooski, VT, USA) was done to determine AGE levels. Intensity of fluorescence was read at emission and excitation wavelengths of 430 nm and 370 nm, correspondingly. Assessment of IL-17A levels was done using standard kits (MILLIPLEX-map-kit, Human cytokine/chemokine panel, Millipore, Billerica, MA, USA), which were used according to manufacturers’ instructions. Flow cytometry was performed as per the protocol described by Liukkonen et al. [34] All samples were assessed in triplicates for AGE and IL-17A levels. Levels of AGE and IL-17A were expressed in micrograms per milliliter (μg/ml) and picograms per milliliter (pg/ml), respectively. Levels of AGE and IL-17A were measured by a blinded and calibrated investigator (Kappa score 0.88).

### Power and statistical analyses

Sample-size estimation (SSE) was done on data from a pilot investigation. Power analysis was done using the G*Power 3.1.9.7 program at a 95% confidence level and power factor of 85%. The primary parameters for SSE were PD and CBL in the study groups. According to the results of SSE, it was estimated that with an alpha of 1%, inclusion of at least 20 individuals per group would be needed to detect a 2 mm difference in PD and CBL in the study groups and attain a power of 85% after considering 15% dropouts. Statistical analyses were done using the independent t- and Wilcoxon rank-sum tests. Logistic regression analysis was done to determine correlation of salivary AGE and IL-17A with age, gender, peri-implant clinicoradiologic parameters, mode of prosthesis retention (cement versus screw retention) and duration of implants in function. Statistical significance was marked for P-values that were below 0.01. The statistician was blinded to the study groups.

## Results

### Response to invitation

**Fig. 1:**
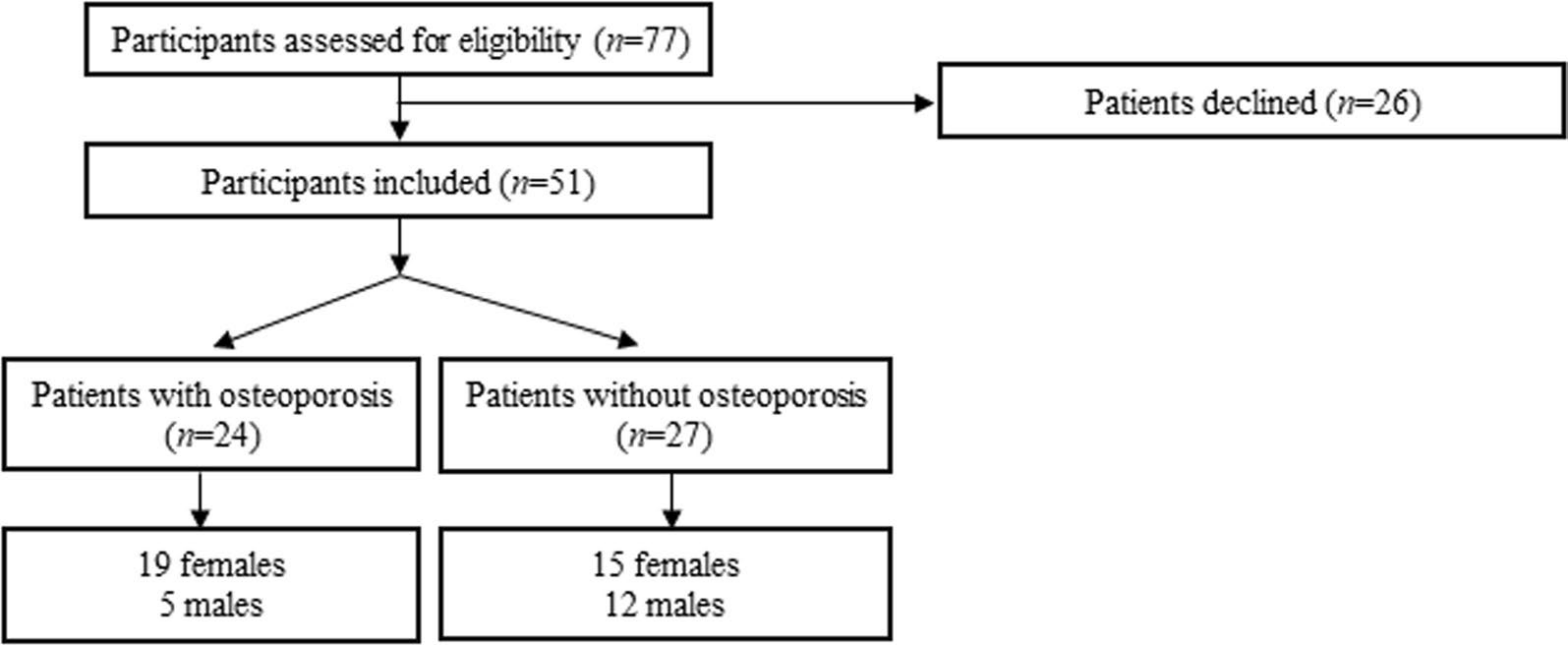
Patient recruitment protocol

Written invitation letters were posted to 77 patients (36 and 41 individuals with and without a diagnosis of osteoporosis, respectively). Twenty-six individuals did not respond to the invitation. Fifty-one individuals (24 and 27 with and without osteoporosis, respectively) signed the informed consent form and agreed to participate in the present study. Among patients with and without osteoporosis, 19 and 15 patients, respectively were females (Fig. 1).

### Questionnaire

All participants (n = 51) were Asian-Indian in origin. There was no significant difference in the mean ages of patients with (67.3 ± 5.1 years) and without (63.4 ± 2.8 years) osteoporosis. Among patients with osteoporosis, number of females were significantly higher than males (*P* < 0.01). There was no significant difference in gender among individuals without osteoporosis. Toothbrushing twice daily was reported by 100% patients with and 81.5% patients without osteoporosis. Flossing of full mouth interproximal spaces once daily was reported by 100% and 77.8% patients with and without osteoporosis, respectively. Seventy-five percent of patients with osteoporosis were receiving dental prophylaxis every four months. Six individuals (25%) with osteoporosis reported that they were visiting a hygienist biannually. In patients without osteoporosis. 66.7% and 33.3% individuals were receiving professional dental prophylaxis by a hygienist every 4 and 6 months, respectively (Table 1). All patients with osteoporosis had been prescribed vitamin supplementation, exercises and oral bisphosphonates by their healthcare providers.

**Table 1:** Demographics of patients with osteoporosis and controls

Parameters	Patients with osteoporosis	Patients without osteoporosis (controls)	*P* value
Patients (*n*)	24 patients	27 patients	0.25
Ethnic origin	Asian–Indian	Asian–Indian	NA
Male (*n*)	5 patients	12 males	*P* < 0.01
Female (*n*)	19 patients	15 females	*P* = 0.16
^*^Mean age in years	67.3 ± 5.1 years	63.4 ± 2.8 years	*P* = 0.2
*Daily toothbrushing*			
Once (%)	0	2	NA
Twice (%)	24 (100%)	22 (81.5%)	*P* = 0.27
*Interproximal flossing*			
Once daily (%)	24 (100%)	21 (77.8%)	*P* = 0.26
Twice daily (%)	0	1 (3.7%)	NA
Never	0	7 (18.5%)	NA
*Dental prophylaxis*			
Every 4 months (%)	18 (75%)	0	NA
Every 6 months (%)	6 (25%)	18 (66.7%)	*P* = 0.28
Every 12 months (%)	0	9 (33.3%)	NA

*NA* Not applicable

### Implants

In both groups, bone level and platform-switched implants were placed in the region of missing teeth in the anterior or posterior maxilla and/or mandible. The diameters and lengths of all implants ranged between 4–5 mm and 11–14 mm, respectively. All implants had been placed at a standardized insertion torque ranging between 30 and 35 Ncm; and 42.4% and 51.7% in patients with and without osteoporosis, respectively were restored with cement-retained restorations. In patients with and without osteoporosis, implants were in function for comparable durations (Table 2).

**Table 2:** Characteristics of implants and duration for which, they were in function

Parameters	Patients with osteoporosis	Patients without osteoporosis (controls)	*P* value
Implants (*n*)	33	29	0.22
Anterior maxilla	2 (6.1%)	4 (13.8%)	0.25
Anterior mandible	3 (9.1%)	2 (6.9%)	0.31
Posterior maxilla^*^	6 (18.2%)	7 (24.1%)	0.26
Posterior mandible^*^	22 (66.6%)	16 (55.2%)	0.19
Implant abutment connection	Platform-switched	Platform-switched	NA
Insertion torque (Newton centimeters)	30 to 35 NCm	30 to 35 NCm	NA
Implant diameter (in millimeters)	4 to 5 mm	4.1 to 5 mm	NA
Implant length (in millimeters)	12 to 14 mm	11 to 14 mm	NA
*Implant prosthesis retention*			
^*^Cement retention (%)	14 (42.4%)	15 (51.7%)	0.31
^†^Screw retention (%)	19 (57.6%)	14 (48.3%)	0.29
^‡^Duration for which, implants were in function (in years)	6.3 ± 0.27 years	6.6 ± 0.5 years	0.23
Depth of implant placement	Bone level	Bone level	NA
Implant supported dentures	None	None	NA
Implant supported dentures	None	None	NA

*NA* Not applicable

*Implants replacing missing premolars or molars

### Peri-implant parameters at six years’ follow-up

None of the implants were lost up to six years’ follow-up (survival rate 100%). There was no significant difference in peri-implant mPI, mBI, PD and mesial and distal CBL around all implants among patients with osteoporosis and controls (Table 3). There was no significant correlation between PD and CBL and whole salivary AGE and IL-17A levels in the study population (Additional file 1: Fig. S1 and Additional file 2: Fig. S2, respectively). There was no correlation between salivary AGE and IL-17A levels and age, gender, duration of osteoporosis, duration of implants in function, jaw location, implant dimensions, mode of implant prosthesis retention, mPI and mBI (data not shown). None of the patients presented with clinical signs of ONJ such as pain and purulent discharge from exposed osseous tissues (Table 3).

**Table 3:** Modified plaque and bleeding indices, probing depth and crestal bone loss around implants

Peri-implant parameters	Patients with osteoporosis	Patients without osteoporosis (controls)	*P* value^*^
*Clinical*			
Modified bleeding index	0.08 ± 0.0006	0.1 ± 0.0003	0.16
Modified plaque index	0.2 ± 0.005	0.3 ± 0.002	0.14
Probing depth (in mm)	1.06 ± 0.04 mm	1.2 ± 0.002 mm	0.11
*Radiologic*			
Crestal bone loss (mesial) (in mm)	0.3 ± 0.005 mm	0.2 ± 0.005 mm	0.18
Crestal bone loss (distal) (in mm)	0.2 ± 0.003 mm	0.3 ± 0.007 mm	0.16

*Statistical comparisons among patients with and without osteoporosis

### Salivary flow rate and AGE and IL-17A levels

In patients with and without osteoporosis, there was no statistically significant difference in flow rate of WS and levels of AGE and IL-17A (Table 4).

**Table 4:** Salivary flow rate and levels of advanced glycation endproducts and interleukin-17

Peri-implant parameters	Patients with osteoporosis	Patients without osteoporosis (controls)	*P* value^*^
Salivary flow rate (ml/min)	0.13 ± 0.04 ml/min	0.12 ± 0.03 ml/min	0.18
Advanced glycation endproduct (μg/ml)	119.6 ± 26.5 μg/ml	91.5 ± 14.6 μg/ml	0.09
Interleukin-17 (pg/ml)	5.1 ± 0.8 pg/ml	4.6 ± 0.3 pg/ml	0.15

*Statistical comparisons among patients with and without osteoporosis

## Discussion

The present observational study was based on the *null* hypothesis that there is no difference in whole salivary AGE and IL-17A levels and peri-implant clinical (PD, mBI and mPI) and radiographic (CBL) status among patients with and without osteoporosis. In summary, results of the present investigation showed that at approximately six years of follow-up scores of mBI, mPI, PD and CBL were statistically insignificant among patients with osteoporosis and controls (patients without osteoporosis). In other word, none of the patients presented with peri-implant diseases (peri-implant mucositis or/and peri-implantitis) when they were clinically and radiologically evaluated at 6 years of follow-up. It is also important to mention that all DI were in function were lost at the follow-up. Moreover, results from laboratory-based investigations showed that whole salivary levels of AGE and IL-17A were comparable and within minimum detection range among individuals without and with osteoporosis. In this regard, the results of the present investigation support the *null* hypothesis; and demonstrated that a diagnosis of osteoporosis does not negatively influence the success and survival of DI. The authors support results presented in the study by Holahan et al. [35] in which, the influence of osteoporosis towards the survival of DI was retrospectively evaluated. In this study, more than 3,224 DI in 746 females aged at least 50 years were assessed [35]. The results showed that patients with osteoporosis were not at an increased risk of implant failure compared with controls [35].

In the current investigation the survival of dental implants in both groups (patients with and without osteoporosis) was 100%. There are a number of factors that seem to have influenced these outcomes. The study population evaluated in the current investigation seemed to be literate towards their oral health. Results obtained from the questionnaire revealed that all patients with osteoporosis were performing flossing of interproximal spaces and teeth brushing teeth once and twice daily. Although percentages for these domestic oral hygiene maintenance protocols were slightly less in controls, they were statistically insignificant compared with osteoporotic patients. Moreover, all patients with osteoporosis and nearly 67% without osteoporosis were receiving professional dental prophylaxis by dental hygienists or dentists biannually. These factors may have helped maintain peri-implant health in both groups. Here, it meaningful to mention that peri-implant health is characterized by features such as absence of (a) soft tissue swelling, (b) gingival redness and bleeding, and (c) CBL following initial healing [36]. All implants assessed in the present investigation fulfilled these criteria. From an immune-inflammatory perspective, the authors claim that routine oral hygiene maintenance not only reduces peri-implant swelling, redness, bleeding and bone loss but also minimizes the expression of salivary biomarkers of inflammation including AGE and IL-17A. The authors support results of a clinical study, in which Al-Amri et al. [37] concluded that as long as oral hygiene is routinely maintained, dental implants can remain clinically and radiologically stable in immunosuppressed patients in a manner similar to their systemically healthy counterparts. Furthermore, none of the participants at ix years’ follow-up demonstrated any clinical signs of ONJ (such as exposure of bone and peri-implant tissue necrosis) in our study. In this context, a diagnosis of osteoporosis is not a contraindication to DI therapy and does not affect their long-term survival as long as oral hygiene is stringently maintained. Similar conclusions were made by Chen et al. [38] and de Medeiros et al. [39].

It has been reported that DI that are placed in tobacco-smokers are more likely to demonstrate signs and symptoms of peri-implant mucositis and peri-implantitis compared with individuals not using any type of tobacco products [21, 40]. Moreover, AGE levels are elevated in PISF of tobacco-smokers than non-smokers [21]. Similarly, from a systemic perspective, metabolic diseases such as poorly-controlled DM pose an increased risk for peri-implant diseases in susceptible patients [12, 41]. According to Romana and Li-Yu [42] the prevalence type-2 DM in patients with osteoporosis is approximately 22%. Therefore, it is strongly suggested that patients with osteoporosis should be screened for DM. In the present study, patients with self-reported systemic diseases other than osteoporosis (such as DM) were excluded; however, their glycemic levels were not screened using traditional tests such as assessment of fasting plasma glucose or hemoglobin A1c levels. Nevertheless, upon evaluation of the patients’ medical records, none of the patients that agreed to participate in the present study had a diagnosis of systemic diseases such as DM, respiratory conditions and/or CD. It is likely that osteoporotic patients with impaired glycemic levels (hyperglycemia) are an increased risk of developing peri-implant diseases compared with non-diabetic individuals with osteoporosis. This warrants additional studies as previously recommended in a meta-analysis by Chen et al. [38] Another limitation of the present investigation is that self-reported tobacco-product consumers (such as cigarette smokers) were not sought. Tobacco-smoking is an independent risk factor of low BMD and bone loss around teeth and DI [31, 43–45]. It is hypothesized that salivary levels of AGE and IL-17A are high and periodontal and peri-implant clinicoradiographic signs are worse in diabetic patients with osteoporosis and smokers with impaired BMD. Further studies are required to test this hypothesis.

## Conclusion

Within the limitations of the present study osteoporotic patients are not at an increased risk of peri-implant diseases and can demonstrate salivary AGE and IL-17A levels comparable to non-osteoporotic individuals as long as oral hygiene is stringently maintained.

## Supplementary Information

**Additional file 1: Fig. S1. **Correlation between levels of advanced glycation endproducts and peri-implant probing depth and clinical attachment loss in the study population.

**Additional file 2: Fig. S2.** Correlation between levels of Interleukin-17A and peri-implant probing depth and clinical attachment loss in the study population. Correlation between PD and CBL and whole salivary AGE and IL-17A levels in the study population.

## Data Availability

The research data and/or materials are not publicly available as the authors did not entail consents to publish this data; however, the data is available from the corresponding author upon reasonable request.
