# Supplementary figures and images for: Levels of whole salivary advanced glycation end products and interleukin-17 and peri-implant clinical and radiographic status in patients with osteoporosis at 6-years’ follow-up
Source: BMC Oral Health. 2022 Nov 24;22:526. doi: 10.1186/s12903-022-02591-7 (PMC9685916; doi:10.1186/s12903-022-02591-7)

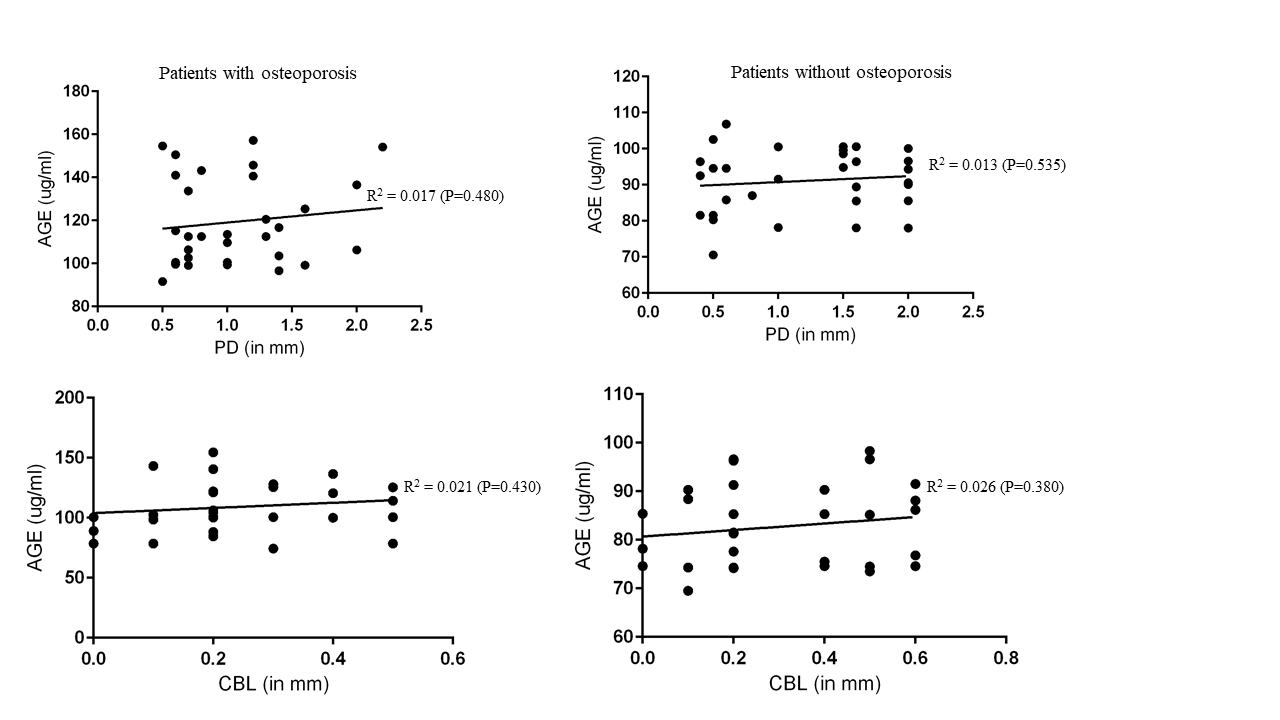

Supplement: Supplementary file 1 — Additional file 1: Fig. S1. Correlation between levels of advanced glycation endproducts and peri-implant probing depth and clinical attachment loss in the study population. [file 12903_2022_2591_MOESM1_ESM.tif]

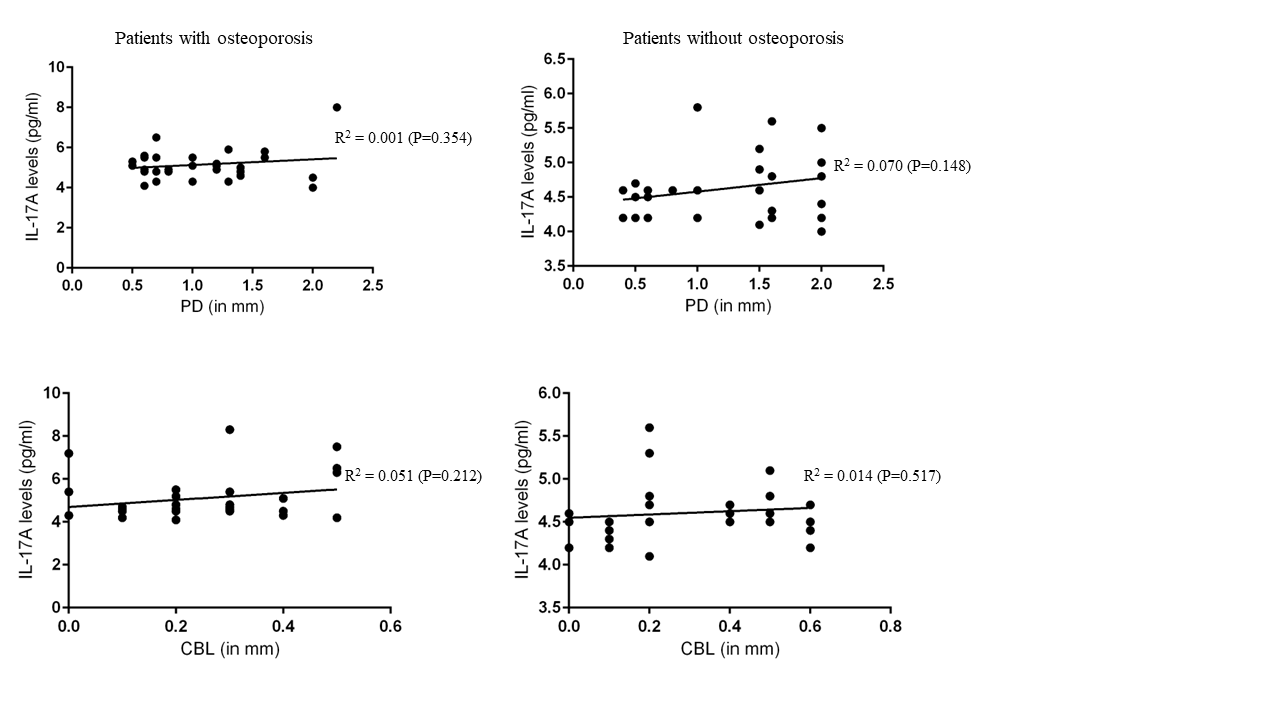

Supplement: Supplementary file 2 — Additional file 2: Fig. S2. Correlation between levels of Interleukin-17A and peri-implant probing depth and clinical attachment loss in the study population. Correlation between PD and CBL and whole salivary AGE and IL-17A levels in the study population. [file 12903_2022_2591_MOESM2_ESM.tif]
